# 11β-HSD1 determines the extent of muscle atrophy in a model of acute exacerbation of COPD

**DOI:** 10.1152/ajplung.00009.2022

**Published:** 2023-02-21

**Authors:** Justine M. Webster, Kelsy Waaijenberg, Wouter R. P. H. van de Worp, Marco C. J. M. Kelders, Sara Lambrichts, Claire Martin, Frank Verhaegen, Brent Van der Heyden, Charlotte Smith, Gareth G. Lavery, Annemie M. W. J. Schols, Rowan S. Hardy, Ramon C. J. Langen

**Affiliations:** ^1^Institute of Inflammation and Ageing, University of Birmingham, Birmingham, United Kingdom; ^2^Institute of Metabolism and Systems Research, University of Birmingham, Birmingham, United Kingdom; ^3^MRC Arthritis Research UK Centre for Musculoskeletal Ageing Research, University of Birmingham, Birmingham, United Kingdom; ^4^Centre for Endocrinology, Diabetes and Metabolism, Birmingham Health Partners, Birmingham, United Kingdom; ^5^Faculty of Health, Medicine and Life Sciences, Department of Respiratory Medicine, NUTRIM School of Nutrition and Translational Research in Metabolism, Maastricht University, Maastricht, The Netherlands; ^6^Institute of Clinical Sciences, University of Birmingham, Birmingham, United Kingdom; ^7^Department of Radiation Oncology (MAASTRO), GROW—School for Oncology and Developmental Biology, Maastricht University Medical Centre, Maastricht, The Netherlands; ^8^Department of Biosciences, Nottingham Trent University, Nottingham, United Kingdom

**Keywords:** 11 β-hydroxysteroid dehydrogenase type 1, COPD, glucocorticoids, inflammation, muscle atrophy

## Abstract

Muscle atrophy is an extrapulmonary complication of acute exacerbations (AE) in chronic obstructive pulmonary disease (COPD). The endogenous production and therapeutic application of glucocorticoids (GCs) have been implicated as drivers of muscle loss in AE-COPD. The enzyme 11 β-hydroxysteroid dehydrogenase 1 (11β-HSD1) activates GCs and contributes toward GC-induced muscle wasting. To explore the potential of 11βHSD1 inhibition to prevent muscle wasting here, the objective of this study was to ascertain the contribution of endogenous GC activation and amplification by 11βHSD1 in skeletal muscle wasting during AE-COPD. Emphysema was induced by intratracheal (IT) instillation of elastase to model COPD in WT and 11βHSD1/KO mice, followed by vehicle or IT-LPS administration to mimic AE. µCT scans were obtained prior and at study endpoint 48 h following IT-LPS, to assess emphysema development and muscle mass changes, respectively. Plasma cytokine and GC profiles were determined by ELISA. In vitro, myonuclear accretion and cellular response to plasma and GCs were determined in C2C12 and human primary myotubes. Muscle wasting was exacerbated in LPS-11βHSD1/KO animals compared with WT controls. RT-qPCR and western blot analysis showed elevated catabolic and suppressed anabolic pathways in muscle of LPS-11βHSD1/KO animals relative to WTs. Plasma corticosterone levels were higher in LPS-11βHSD1/KO animals, whereas C2C12 myotubes treated with LPS-11βHSD1/KO plasma or exogenous GCs displayed reduced myonuclear accretion relative to WT counterparts. This study reveals that 11β-HSD1 inhibition aggravates muscle wasting in a model of AE-COPD, suggesting that therapeutic inhibition of 11β-HSD1 may not be appropriate to prevent muscle wasting in this setting.

## Graphical abstract

**Figure:**
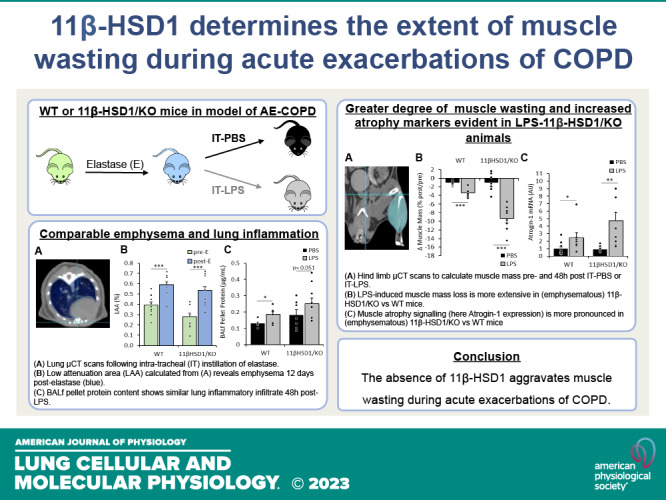

## INTRODUCTION

Chronic obstructive pulmonary disease (COPD), characterized by remodeling of the airways (bronchitis) and destruction of the lung parenchyma (emphysema), is predicted to be the third leading cause of death worldwide by 2030 (1). Skeletal muscle wasting is a severe extrapulmonary complication of COPD, particularly in patients with emphysema (2, 3), which contributes to frailty and poor disease outcomes, and is an independent predictor of mortality (4). To date, therapeutic strategies designed to prevent or reverse the acute process of muscle wasting in COPD patients are limited, and primarily restricted to exercise and nutritional intervention, with limited efficacy in the acute exacerbation disease phase (5, 6).

Acute exacerbations of COPD (AE-COPD), typically secondary to acute pulmonary and systemic inflammation with infection, accelerate muscle loss in COPD (7). Glucocorticoids (GCs), such as cortisol (corticosterone in mice), are a class of endogenous anti-inflammatory steroids that are elevated in response to systemic inflammation (8). This increase in circulating cortisol is mediated by the inflammatory activation of the hypothalamic-pituitary-adrenal axis (HPA) resulting in increased adrenal output (8). Synthetic therapeutic glucocorticoids possess potent anti-inflammatory immunomodulatory properties and are frequently utilized in the management of COPD patients during inflammatory exacerbation (9). Both elevated endogenous and therapeutic GCs are associated with increased GC signaling within skeletal muscle (10), and contribute to muscle atrophy in human disease, through the activation of proteolytic pathways, and suppression of anabolic signaling and myonuclear turnover (11–13).

The enzyme 11 β-hydroxysteroid dehydrogenase type 1 (11β-HSD1) converts inactive endogenous GCs precursors (cortisone in humans, 11-dehydrocorticosterone in mice) into their active form (cortisol and corticosterone respectively), where it amplifies GC signaling and determines peripheral tissue exposure to GCs (14). Its expression is potently upregulated in muscle in response to proinflammatory mediators present during acute exacerbation of COPD (including cytokines such as IL-1β and TNF-α), as well as by alternative factors implicated in COPD muscle wasting such as hypoxia and fasting (15–17). Several studies have revealed a critical role for 11β-HSD1 in determining local endogenous and therapeutic GC levels within the muscle, which in turn modulate anti-anabolic and catabolic muscle metabolism in the primary cell and ex vivo tissue cultures and in vivo models (15, 16). However, although therapeutic inhibitors of 11β-HSD1 have been widely explored in Phase II clinical trials in the management of metabolic diseases such as hypertension, osteoporosis, and insulin resistance, their application in the management of muscle wasting in inflammatory diseases such as COPD remains poorly defined (18–25).

The objective of this study was to ascertain the contribution of endogenous corticosteroid activation and amplification by 11β-HSD1 toward the pathophysiology of skeletal muscle wasting during an acute exacerbation of emphysematous-COPD and test the hypothesis that 11β-HSD1 inhibition can prevent GC induced muscle wasting in this context.

To achieve this, we used a murine model of AE-COPD with global transgenic deletion of 11β-HSD1 (11βHSD1/KO), in combination with in vitro approaches to assess the contribution of circulating endocrine mediators by exposing cultured muscle cells to plasma of WT and 11β-HSD1/KO AE-COPD mice. Our data show aggravated skeletal muscle wasting during AE-COPD in mice with transgenic deletion of 11β-HSD1 compared with wild-type counterparts. This coincides with an elevation of circulating endogenous GC levels and an attenuation of anabolic recovery mechanisms in these 11βHSD1/KO animals following the acute exacerbation. These data offer new insights into the role of endogenous GCs metabolism in muscle atrophy and the efficacy of targeting 11β-HSD1 in the management of muscle wasting in AE-COPD. This study shows that 11β-HSD1 inhibition does not prevent but aggravates muscle wasting during an acute exacerbation of COPD.

## MATERIALS AND METHODS

### Animal Models

To determine the role of 11β-HSD1 in skeletal muscle wasting during an acute exacerbation of COPD, WT, and global 11β-HSD1 knockout (11βHSD1/KO) mice were used. Global 11βHSD1/KO mice were achieved using Cre-loxP technology, generating a Tri-loxed 11β-HSD1 allele by flanking exon 5 with LoxP sites, as previously described and maintained on a C57BL/6J background (26). Male WT and 11βHSD1/KO mice (aged 14–20 wk, *n* = 33) received 2 weekly intratracheal instillations (IT; *D0*, *D7*), with 3 U of porcine pancreas elastase (E; Elastin Products Company, Missouri) dissolved in 50 μL of sterile PBS^−/−^ to induce emphysema. The later adult stage of development was selected so as to remove complications in the interpretation of data arising from high anabolic growth evident at earlier stages of development. (27). Presence of emphysema was determined by micro–Computed Tomography (μCT) analysis (*D12*). WT and 11βHSD1/KO animals were then divided randomly into two subgroups (*n* = 7–9), receiving a single bolus (IT) of 2 μg/g mouse of LPS (*Escherichia coli*, 055:B5, Sigma, Dorset, UK) dissolved in sterile PBS^−/−^ or PBS^−/−^ alone (v.c.) to evoke a pulmonary inflammatory response (AE) (28, 29). Animals were euthanized 48 h post-LPS or PBS IT-instillation and hind limb muscles [gastrocnemius, soleus, tibialis anterior, extensor digitorum longus (EDL), and plantaris] dissected, weighed, and snap frozen in liquid nitrogen and stored at −80°C for biochemical analysis. Bloods were collected from abdominal vena cava to obtain plasma. Right lungs lobes were rinsed with Hank’s balanced salt solution (HBSS) to obtain bronchoalveolar lavage fluid (BALf), left lung lobes were collected and snap frozen for biochemical analysis. All animals were socially housed (*n* = 2–4/cage) in standard conditions (12 h/12h dark-light cycle, temp 21 ± 1°C) with ad libitum access to standard chow and water. All experiments were carried out at the central animal facility at Maastricht University. Protocols and procedures involving mice were approved by the Institutional Animal Care Committee of Maastricht University and the Central authority for scientific procedures on animals (CCD; AVD1070020198766). The group size was determined by power calculations based on previous experience with this model. All analyses derived from animal experiments were analyzed blinded.

### μCT Imaging and Assessment of Emphysema and Muscle Mass

Animals were anesthetized using a mixture of air and isoflurane (4% induction, 2% maintenance) and scanned using a micro cone beam CT (µCBCT) scanner (XRAD-225Cx, Precision X-Ray, North Branford) at an X-ray tube potential of 50 kVp and an X-ray current of 5.6 mA, giving an imaging dose of 0.3 Gy. The µCBCT projection data were reconstructed using the Feldkamp back-projection algorithm with a voxel dimension of 100 × 100 × 100 µm^3^ (30). A mouse-mimicking CT calibration phantom with 12 cylindrical tissue-mimicking inserts of 3.5 mm diameter (SmART Scientific Solutions, Maastricht, the Netherlands) (31) was used to also calibrate the µCBCT scanner in mass density (g/cm^3^) units. Next, the lung volumes were manually segmented on every reconstructed µCBCT using the SmART-ATP software (version 1.3.6, Precision X-ray Inc.) (32), and the density of every segmented lung voxel was plotted in a mass density histogram. Low attenuation area (LAA) threshold was set from −871 (0.21 g/cm^3^) to −610 (0.45 g/cm^3^) Hounsfield units (HU). Muscle volumes were established using the convolutional neural network as previously described (33).

### Lung Histology

Lungs were fixed in formalin and embedded in paraffin. Sections 4 μm in thickness were cut with a microtome. Slides were dewaxed with xylene, rehydrated, and stained with Mayer’s hematoxylin and eosin (both VWR International B.V.). Stained slides were dehydrated and mounted with glass coverslips using a xylene-based mounting medium (DPX, Sigma-Aldrich). The sections were examined by light microscopy using a Nikon Eclipse E800 microscope (Nikon Instruments Inc.) with ZEN 3 software (Carl Zeiss Microscopy GmbH) to assess inflammatory infiltrate.

### ELISA Analysis

Plasma corticosterone (R&D Systems Parameter KGE009, Minneapolis) and IL-6 (R&D Systems Quantikine M6000B, Minneapolis) levels were determined using a commercially available ELISA assay in accordance with the manufacturer’s instructions, and optical density was determined at 450 nm using a microplate reader.

### RNA Isolation and Analysis of Gene Expression

Lung and muscle (gastrocnemius) RNA was extracted by mechanical suspension and lysis of powdered tissue in TRIzol reagent (Sigma-Aldrich Chemie B.V., the Netherlands). Phase separation and RNA precipitation were performed with the addition of isopropanol (Sigma-Aldrich Chemie B.V., the Netherlands) and glycogen (Invitrogen 10814). RNA precipitates were washed in 70% ethanol and reconstituted in RNA storage solution (Invitrogen AM700) and stored at −80°C.

Lung cDNA was generated using the Tetro cDNA Synthesis Kit (GC biotech) according to the manufacturer’s instructions. Expression of specific genes was assessed by real-time Polymerase chain reaction (PCR) on a LC480 software (v. 2014.1) and relative DNA starting quantities of the samples were derived using LinRegPCR software (v. 2014.0, Ruijter). Expression of genes of interest (Table 1) was normalized using GeNorm software by geometric average of three reference genes (Cyclophilin, RPLP0, and YWHAZ).

**Table 1.** Sequence of primers used for RT-qPCR to assess gene expression

Gene	Forward Primer (5′ to 3′)	Reverse Primer (5′ to 3′)
*Cyclophilin*	TTCCTCCTTTCACAGAATTATTCCA	CCGCCAGTGCCATTATGG
*RPLP0*	GGACCCGAGAAGACCTCCTT	GCACATCACTCAGAATTTCAATGG
*YWHAZ*	TGCTGGTGATGACAAGAAAGGAA	AACACAGAGAAGTTGAGGGCCA
*CXCL2*	CCCTGGTTCAGAAAATCATCCAAA	TTTGGTTCTTCCGTTGAGGGAC
*IL-1RA*	GCCAGGACCGCTCAGAGA	TGCCTCGACTGTTAGTCAAGCA
*GILZ*	GGGGCAGAGATGGGAGAGAT	CCCAAAGCTGTAACCCCACA
*FoxO1*	AAGAGCGTGCCCTACTTCAAGGATA	CCATGGACGCAGCTCTTCTC

Muscle cDNA was generated using the Multiscribe reverse transcription kit (Thermo Fisher Scientific, Loughborough, UK) according to the manufacturer’s instructions. Expression of specific genes was assessed by real-time PCR using TaqMan Gene Expression Assays (Thermo Fisher Scientific, Loughborough, UK) on an ABI7500 system (Applied Biosystems, Warrington, UK). Expressions of genes of interest (Thermo Fisher Scientific, Loughborough, UK) Fbxo32 (*Atrogin-1*, Mm00499523), *Trim63* (*Murf-1*, Mm01185221), *CXCL1* (Mm04207460), *IκBα* (Mm004777798), and *IL-1β* (Mm00434228) were normalized using GeNorm software by geometric average of three reference genes (GAPDH, HPRT, and YWHAZ).

All data were expressed as arbitrary units (AU) using the calculation; DDCt = DCt(experimental group) − DCt(control group) and reported as fold-change = 2DDCt.

### Western Blot and Analysis of Protein

Powdered gastrocnemius muscle and lysed in ice-cold IP-lysis buffer containing protease inhibitors (Complete; Roche Nederland, Woerden, the Netherlands), using a rotating blade tissue homogenizer (Polytron homogenizer, Kinematica). The total protein concentration of the supernatant was determined with a BCA protein assay kit (Pierce Biotechnology, Cat. No. 23225, Rockford, IL) according to the manufacturer’s instructions. Alternatively, the cell pellet obtained from BAL fluid was collected following centrifugation (10’, 1,500 *g*, 4°C) and stored at −80°C. Lysates were prepared and processed for Western blot analysis. Proteins were denatured in Laemmli buffer 100°C for 5 min. Ten micrograms of protein were separated on a Criterion XT Precast 4%–12% or 12% Bis-Tris gel (Bio-Rad Laboratories, Veenendaal, the Netherlands) and transferred onto nitrocellulose transfer membrane (Bio-Rad Laboratories). The membrane was stained with Ponceau S solution (0.2% Ponceau S in 1% acetic acid; Sigma-Aldrich Chemie) to control for protein loading. After blocking in 5% nonfat dried milk, membranes were incubated at 4°C overnight with primary antibodies (Table 2). All antibodies were diluted 1:1,000 in Tris Buffered Saline (TBS)-Tween plus 5% Bovine serum albumin (BSA) or 3% skimmed milk. Signal detection used horseradish peroxidase-conjugated secondary antibody (1:10,000 in nonfat fried milk; Vector Laboratories, Burlingame, CA) and visualized with chemiluminescence (Supersignal West Pico or Femto Chemiluminescent Substrate; Pierce). Membranes were imaged (Amersham Imager 600, GE Life Sciences) and quantified using Image Quant software.

**Table 2.** Antibodies used for western blot

Antibody	Cat. No. (Cell Signaling, UK, Unless Otherwise Stated)
p-ULK1 (S757)	6888
ULK1	8054
p-FoxO1 (S256)	9461
FoxO1	2880
p-Akt (S473)	9271
Akt	9272
p-S6 (S235/236)	4858
S6	2217
4E-BP1	9452
MyLC-3 (F310)	AB 531863 (from DSHB, Iowa)
LC3B	2775
F4/80	123102 (from BioLegend, San Diego)

### Assessment of Postnatal Myonuclear Accretion

Blood was centrifuged and plasma was collected. Cre-C2C12 (Cre-IRES-PuroR) cells were differentiated for 4 days, and myotube damage was induced on *day 4* by incubating with HBSS in DM (50%). After 25 h, LV-floxed-Luc (LV-flox-luc) myoblasts were added cells to Cre-C2C12 myoblasts and further incubated with culture medium (5% serum) for 3 days.

Proliferating Cre-C2C12 myoblasts were cultured as above with the absence of myotube damage on *day 4*. On day 5, LV-flox-Luc cells were added to C2C12 myoblasts. Following a 6-h incubation, myotubes were incubated with corticosterone (250 nM; Sigma-Aldrich Chemie B.V, Netherlands, C2505) or dexamethasone (10 μM; Sigma-Aldrich Chemie B.V, Netherlands, D8893) dissolved in DMSO for 3 days.

Luminescence was determined using a luminometer (Berthold Lumat LB9507, Belgium) and corrected for protein content.

### Human Myotube Culture

Reagents were obtained from Sigma (Gillingham, UK) unless stated otherwise. Primary myoblasts obtained from healthy donors (CC‐2580; Lonza, Slough, UK) were maintained in-house in Skeletal Muscle Basal Medium‐2 (Lonza; CC‐3244 and CC‐3246) containing 0.1% human epidermal growth factor, 2% l‐glutamine, 10% fetal bovine serum (FBS), and 0.1% gentamicin/amphotericin‐B in the absence of GCs. Confluent myotubes were differentiated in Dulbecco’s modified Eagle’s medium (DMEM) containing 2% horse serum (HS) for 120 h. Media were replaced every 2–3 days as previously reported (16).

### Statistical Analysis

Data are shown as means ± SE. Comparisons were computed using GraphPad Prism (34). Following the assessment of Gaussian distribution, significance assessment was analyzed by unpaired *t* test, one-way and two-way ANOVA with Tukey’s post hoc analysis or Pearson correlation analysis, or nonparametric equivalent tests as appropriate. Statistical significance was defined as *P* value < 0.05 (*P < 0.05; **P < 0.01; ***P < 0.001; no asterisk or NS, *P* > 0.05).

## RESULTS

### Confirmation of AE-COPD Model in WT and 11βHSD1/KO Mice

**Figure 1.**
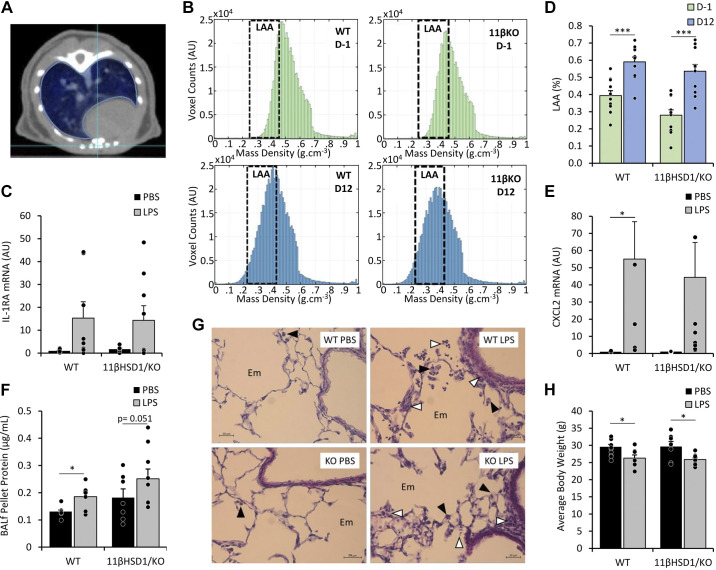
WT and 11β-HSD1/KO mice intratracheally instilled with elastase and LPS developed emphysema and pulmonary inflammation, respectively. μCT scans were obtained 1 day before (*D1*) and 12 days following (*D12*) intratracheal instillation of elastase in WT (*n* = 12) and 11βHSD1/KO (*n* = 11) mice (*A*) and were analyzed for LAA, with a LAA threshold of −871 (0.21 g/cm^3^) to −610 (0.45 g/cm^3^) Hounsfield units (HU; *B*–*D*). Male mice then received a single bolus of PBS or LPS (*n* = 7–9/group) and lungs and BALf collected after 48 h for mRNA and protein analysis. Gene expression (AU) of Lung IL-1RA (*C*) and CXCL2 (*E*) were determined and expressed as fold-change compared with control. Total protein concentrations of BALf pellet (*F*) were determined and expressed as fold-change compared with control. Histological analysis of lung cross-sections (magnification ×400) were analyzed for macrophage and granulocyte infiltration following PBS and LPS treatment in WT and 11βHSD1/KO animals (black arrowhead: alveolar macrophage; white arrowhead: infiltrating granulocyte; *G*). Body weights were taken before euthanasia (*H*) expressed as means ± standard error. Statistical significance was determined using two-way analysis with Tukey’s post hoc analysis and unpaired *t* test. *P < 0.05, ***P < 0.001. AU, arbitrary units; BALf, bronchoalveolar lavage fluid, 11βHSD1/KO, 11βHSD1 global genetic deletion; Em, enlarged alveolar space; LAA, low attenuation area; WT, wild type.

To investigate the role of 11β-HSD1 in muscle wasting during AE-COPD, WT and 11βHSD1/KO mice were intratracheally instilled with elastase, followed by instillation of LPS or vehicle control to evoke a pulmonary inflammatory response in an emphysematous background (Supplemental Fig. S1) (29, 35). To determine emphysema development in both WT and 11βHSD1/KO animals following intratracheal instillation of elastase, μCBCT scans were taken (Fig. 1*A*) and lung density histograms were made for each mouse lung (Fig. 1*B*). In line with the left-ward shift suggesting a decrease in the density of the lung tissue, increased LAA of the lungs on day 12 (*D12*) compared with any prior elastase treatments on *D*–*1* confirmed the presence of emphysema in WT and 11βHSD1/KO mice (fold-change WT = 1.5, *P* < 0.001; 11βHSD1/KO = 1.9, *P* < 0.001), with no significant differences seen between animal genotypes (Fig. 1*D*). Emphysema was also apparent from the enlarged alveolar space observed in all animals (Fig. 1*G*). Subsequently, emphysematous WT and 11βHSD1/KO animals were intratracheally instilled with either LPS, to evoke a pulmonary inflammatory response or PBS. Inflammatory gene expression markers *IL-1RA* (fold-change WT = 15.3, *P* = 0.07; 11βHSD1/KO = 8.2, *P* = 0.07; Fig. 1*C*) and *CXCL2* (fold-change WT = 55, *P* = 0.04; 11βHSD1/KO = 41, *P* = 0.07; Fig. 1*E*) in lung tissue of mice treated with LPS were increased compared with WT counterparts for CXCL2, although only showed statistical significance in WT animals.

In addition, total protein obtained from bronchoalveolar lavage fluid (BALf) pellet increased (WT: *P* ≤ 0.05) or tended to increase (11βHSD1/KO = *P* = 0.051) in response to LPS, suggestive of inflammatory cell infiltration in the lumen of the lungs (Fig. 1*F*). This was supported by an observed increase in levels of the macrophage marker F4/80 in BALf cell pellets (Supplemental Fig. S2, *A* and *B*), although this was not significant in 11βHSD1/KO, and increased alveolar macrophage and granulocyte infiltration apparent in lung tissue of WT and 11βHSD1/KO animals treated with LPS (Fig. 1*G*).

To assess the systemic impact of AE-COPD, body weights were assessed 48 h postinstallation (Fig. 1*H*). Both WT and 11βHSD1/KO animals showed a significant reduction in body weights following LPS treatment compared with PBS controls (−12%, *P* < 0.05 and −13%, *P* < 0.05, respectively; Supplemental Fig. S3*B*), with no significant differences seen between LPS-treated WT and 11βHSD1/KO animals.

Combined, these data demonstrate that the lung inflammatory response and body weight loss as a systemic consequence of AE-COPD are preserved in emphysematous 11βHSD1/KO mice.

### Muscle Wasting Is More Pronounced in LPS-Treated Emphysematous 11βHSD1/KO Mice

**Figure 2.**
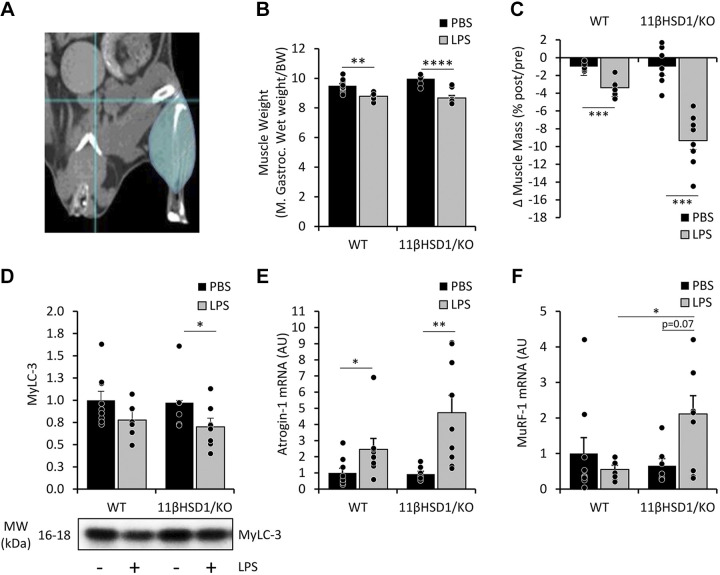
LPS-treated emphysematous 11βHSD1/KO mice show exacerbated muscle wasting compared with WT controls. μCT scans were taken pre- and posttreatment of LPS for 48 h in male WT and 11βHSD1/KO mice (*n* = 7–9/group; *A*) and changes in muscle mass of total hind limb (*C*) were determined. Gastrocnemius wet muscle weights were measured and expressed relative to the start body weight (*B*). Protein levels of MyLC-3 (*D*) were assessed, normalized to Tubulin, and expressed as fold-change compared with control with representative western blot images. Gene expression (AU) of homogenized gastrocnemius Atrogin-1 (*E*) and MuRF-1 (*F*) levels were determined and expressed as fold-change compared with control. Values are expressed as means ± standard error. Statistical significance was determined using two-way analysis with Tukey’s post hoc analysis and unpaired *t* test. *P < 0.05, **P < 0.005, ***P < 0.001, ****P < 0.0001. 11βHSD1/KO, 11βHSD1 global genetic deletion; BW, body weight; M. Gastroc., gastrocnemius; WT, wild type.

To investigate the involvement of 11β-HSD1 in the impact of lung inflammation on muscle wasting in this model (28, 29), M. gastrocnemius wet weights were assessed, showing a significant reduction in LPS-treated compared with PBS control animals of both genotypes (−7%, *P* < 0.005 and −13%, *P* < 0.0001 respectively; Fig. 2*B*). In addition, hind limb muscle mass was calculated using noninvasive μCBCT scans (Fig. 2, *A* and *C*) of hind limb muscles 48 h post-LPS instillation (Supplemental Fig. S3*B*) Importantly, calculating individual muscle mass changes derived from μCBCT scans obtained from animals pre- and post-PBS/LPS instillation, revealed a stronger reduction in muscle mass WT compared with 11βHSD1/KO animals treated with LPS (−3.4% vs. −9.3%, *P* < 0.0005; Fig. 2*C*).

Accordingly, a significant decrease (*P* < 0.05) in MyLC3 protein content was observed following LPS in the muscle of 11βHSD1/KO mice, whereas in WT mice only a trend toward a decrease (*P* = 0.09) was observed (Fig. 2*D*). To determine the effects of AE-COPD on muscle wasting in WT and 11βHSD1/KO animals we examined *Atrogin-1* and *MuRF-1* expression levels as these E3 ligases are involved in muscle proteolytic responses (36). LPS treatment significantly induced *Atrogin-1* mRNA levels in the gastrocnemius muscle after 48 h in both WT and 11βHSD1/KO mice (fold-change WT = 2.5, *P* < 0.05; 11βHSD1/KO = 5.1, *P* < 0.005; Fig. 2*E*). Although the LPS-induced increase in *Atrogin-1* mRNA levels appeared more pronounced (∼ twofold) in the muscle of 11βHSD1/KO compared with WT animals, this was not significantly different. In contrast, only in 11βHSD1/KO muscle *MuRF-1* levels tended to increase in response to LPS (fold-change 11βHSD1/KO = 3.2, *P* = 0.07), whereas no changes were present in the muscle of WT animals (Fig. 2*F*). Combined, these data show more pronounced muscle wasting in 11βHSD1/KO animals following AE-COPD.

### Suppression of Catabolic Signaling Is Reduced in the Muscle of 11βHSD1/KO Compared with WT Animals in Response to LPS

**Figure 3.**
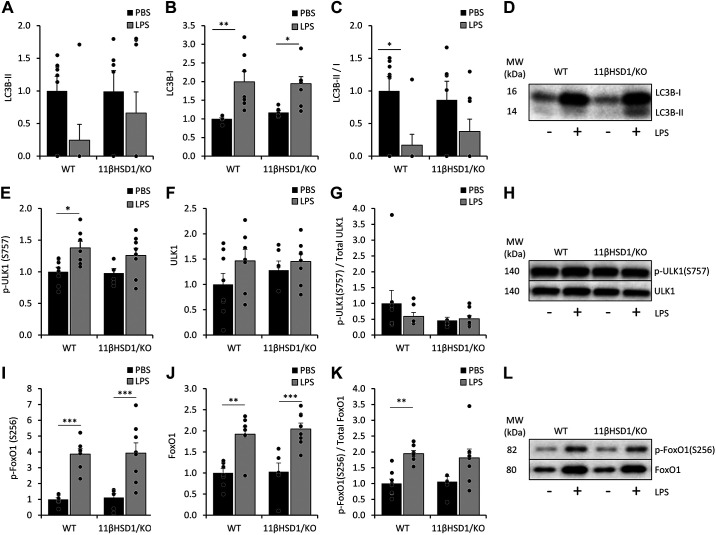
Catabolic signaling in the muscle of WT and 11βHSD1/KO animals in response to LPS. 48 h after PBS or LPS treatment in male WT and 11βHSD1/KO mice (*n* = 7–9/group), gastrocnemius muscle was collected for mRNA and protein analysis. Gastrocnemius protein levels of LC3B-II (*A*) and LC3B-I (*B*) were assessed, normalized to Tubulin, and expressed as fold-change compared with control, with representative western blot images (*D*). Phosphorylated ULK1(S575) (*E*) and FoxO1(S256) (*I*), and total ULK1 (*F*) and FoxO1 (*J*) were assessed, normalized to Ponceau staining and expressed as fold-change compared with control with representative western blot images (*H* and *L*). Ratios LC3B-II/C3B-I (*C*), and phosphorylated and total ULK1 (*G*) and FoxO1 (*K*) were assessed and shown as fold-change compared with control. Statistical significance was determined using two-way analysis with Tukey’s post hoc analysis and unpaired *t* test. *P < 0.05, **P < 0.005, ***P < 0.001. AU, arbitrary units; 11βHSD1/KO, 11βHSD1 global genetic deletion; WT, wild type.

For further insight into catabolic processes in the gastrocnemius muscle, we examined protein levels and phosphorylation status of markers of the autophagy lysosomal pathway using Western blot analysis (Fig. 3, *A*–*L*). LC3B lipidation, resulting in an altered migration pattern (LC3B-II), is an important step in autophagy. Although LC3B-II abundance was not significantly altered (Fig. 3*A*), LC3B-I levels were significantly elevated following LPS in both WT and 11βHSD1/KO mice (Fig. 3*B*). Importantly, the LC3BII/I ratio, indicative of autophagic flux (37), was significantly reduced in LPS-treated WT but not 11βHSD1/KO animals (Fig. 3, *A*–*D*). In addition, ULK1 (Ser757) phosphorylation inhibits autophagosome formation (38), and was increased in WT but not 11βHSD1/KO mice in response to LPS (fold-change WT = 1.4, *P* < 0.05; 11βHSD1/KO = 1.3, *P* = 0.193; Fig. 3*E*), suggesting attenuation of autophagic signaling in WT animals although this increase was not reflected in p-ULK1/Total ULK1 ratios (Fig. 3*G*). The transcriptional activity of FoxO1 is also involved in control of autophagy and is inactivated through phosphorylation on Ser256 (39). Treatment with LPS increased phosphorylation of FoxO1 (Ser256; fold-change WT = 3.9 *P* < 0.001; 11βHSD1/KO = 3.5 *P* < 0.001; Fig. 3*I*) and Total FoxO1 (fold-change WT = 1.9 *P* < 0.005; 11βHSD1/KO = 2 *P* < 0.001; Fig. 3*J*) protein levels across both groups. Despite this, FoxO1 p/total ratio clearly increased in WT (fold-change WT = 1.9 *P* < 0.005) or tended to increase in 11βHSD1/KO mice (fold-change 1.7 *P* = 0.057) following LPS treatment (Fig. 3*K*).

Combined, these data show a coherent suppression of catabolic autophagy signaling following LPS, which is apparent at multiple levels in muscle of WT animals but not in 11βHSD1/KO mice.

### Anabolic Signaling Is Activated in the Muscle of WT Compared with 11βHSD1/KO Emphysematous Mice Following LPS

**Figure 4.**
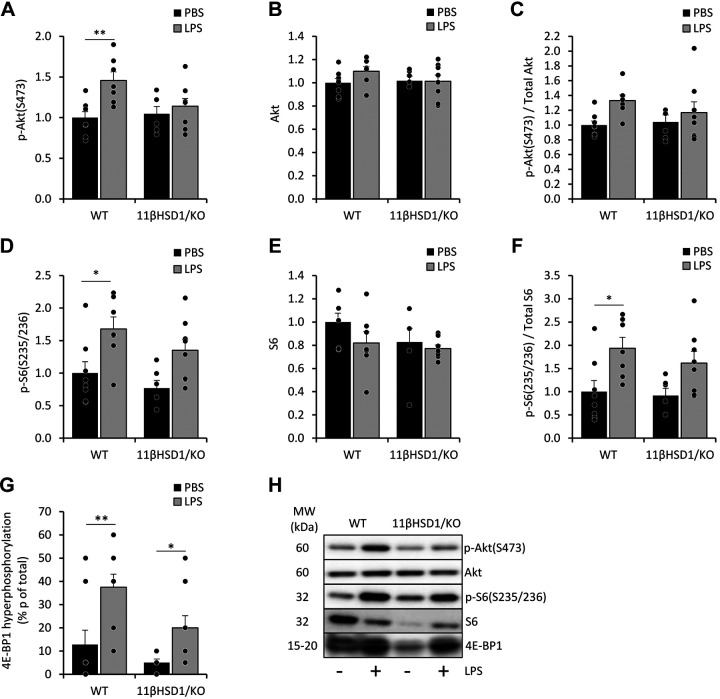
Anabolic signaling in the muscle of WT and 11βHSD1/KO animals in response to LPS. 48 h after PBS or LPS treatment in male WT and 11βHSD1/KO mice (*n* = 7–9/group), gastrocnemius muscle was collected for mRNA and protein analysis. Gastrocnemius protein levels of phosphorylated Akt (S475) (*A*) and S6 (s235/236) (*D*), and total Akt (*B*) and S6 (*E*) were assessed, normalized to Ponceau staining and expressed as fold-change compared with control with representative western blot images (*H*). Ratios of phosphorylated and total Akt (*C*) and S6 (*F*) were assessed and shown as fold-change compared with control. Hyper-phosphorylation of 4E-BP1 was assessed (*G*). Statistical significance was determined using two-way analysis with Tukey’s post hoc analysis and unpaired *t* test. *P < 0.05, **P < 0.005. AU, arbitrary units; 11βHSD1/KO, 11βHSD1 global genetic deletion; WT, wild type.

We next determined whether recovery mechanisms following of AE-COPD-induced muscle atrophy extended to anabolic signaling in the gastrocnemius muscle of WT and 11βHSD1/KO animals. Akt was investigated as an upstream regulator of protein synthesis (Fig. 4, *A*–*C* and *H*) (39). In LPS-treated WT mice, a significant increase in phosphorylation of Akt was observed (fold-change = 1.5, *P* < 0.005; Fig. 4*A*), which was not apparent in 11βHSD1/KO animals. Although this differential increase was also observed for p/total Akt ratio, it was not significant (fold-change =1.3, *P* = 0.118; Fig. 4*C*) due to small changes in total Akt levels (Fig. 4*B*). In addition, phosphorylation status of S6 (Ser235/236), which determines mRNA translation in protein synthesis, was examined (Fig. 4, *D*–*F*, and *H*). Both WT and 11βHSD1/KO animals treated with LPS showed increases in p-S6, with the former having a significant increase (fold-change WT = 1.7, *P* < 0.005; 11βHSD1/KO = 1.7, *P* = 0.112; Fig. 4*D*). This pattern also apparent for p/total S6 ratios (fold-change WT = 1.9, *P* < 0.05; 11βHSD1/KO = 1.8, *P* = 0.187; Fig. 4*F*) and hyper-phosphorylation of 4E-BP1 (fold-change WT = 2.9, *P* < 0.01; 11βHSD1/KO = 4, *P* < 0.05; Fig. 4, *G*–*H*).

Taken together, these data may suggest that 11βHSD1/KO mice treated with LPS have a delayed reactivation of protein synthesis signaling and reduced initiation of a recovery response in skeletal muscle compared with WT counterparts.

### Plasma Corticosterone Levels Are Increased in LPS Treated 11βHSD1/KO Animals and Suppress In Vitro Myonuclear Accretion

**Figure 5.**
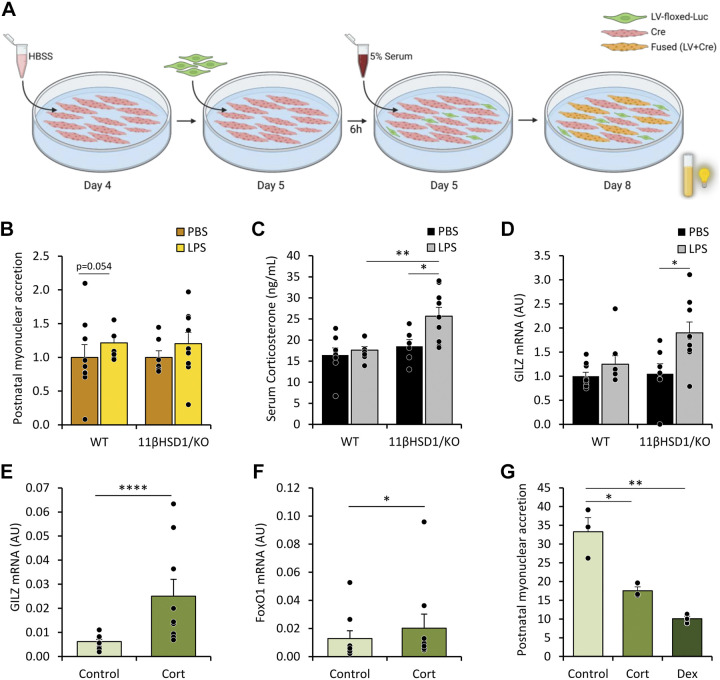
Glucocorticoid levels in plasma and muscle LPS treated WT and 11βHSD1/KO animals and in vitro analysis of myonuclear accretion when treated with glucocorticoids. In vitro postnatal myonuclear accretion of damaged C2C12 myotubes incubated with mouse plasma (*n* = 7–9/group) was determined by luciferase activity (*A* and *B*). Plasma corticosterone levels determined by ELISA (*C*; *n* = 7–9/group). Gene expression (AU) of GILZ from homogenized gastrocnemius expressed as fold-change compared with control (*D*). Gene expression (AU) of GILZ (*E*) and FoxO1 (*F*) of in vitro human primary myotubes treated with cortisol (100 nM; *n* = 3). Effects of corticosterone (250 nM) or dexamethasone (10 μM; 72 h) on postnatal myonuclear accretion in vitro (*n* = 3; *G*), relative luciferase activity (RLU/protein). Statistical significance was determined using two-way analysis with Tukey’s post hoc analysis and unpaired *t* test. *P < 0.05, **P < 0.005. AU, arbitrary units; 11βHSD1/KO, 11βHSD1 global genetic deletion; Cort, corticosterone; Dex, dexamethasone; WT, wild type. [Image created with BioRender.com and published with permission.]

To investigate whether altered muscle recovery responses in WT and 11βHSD1/KO mice reflect the actions of circulating mediators, we examined the impact of the plasma of these animals in a C2C12 model of myonuclear accretion, a process essential for skeletal muscle growth and repair. Mouse plasma was added to damaged C2C12-Cre myotubes cocultured with LV-flox-Luc C2C12 myoblasts and postnatal myonuclear accretion was assessed (Fig. 5*A*). Only in cultures incubated with the plasma of LPS-treated WT mice a trend to significant increase (*P* = 0.051) in myonuclear accretion was observed (Fig. 5*B*). Instead, plasma corticosterone levels were increased but only in LPS-treated 11βHSD1/KO mice compared with WT controls (fold-change = 1.4, *P* < 0.05; Fig. 5*C*) and direct comparison between LPS treated WT and 11βHSD1/KO mice showed plasma corticosterone levels significantly higher in 11βHSD1/KO than WT animals (fold-change = 1.5, *P* < 0.005). Importantly, increased gastrocnemius mRNA levels of *Gilz*, a glucocorticoid responsive gene, were only observed in LPS-treated 11βHSD1/KO mice (fold-change WT = 1.2 *P* = 0.756; 11βHSD1/KO = 1.8 *P* < 0.05; Fig. 5*D*), indicative of CORT-induced signaling in affected skeletal muscle. Human primary cultures treated with corticosterone showed significant increases in Gilz and FoxO1 mRNA expression (Fig. 5, *E* and *F*). We then examined the impact of elevated corticosterone levels in a murine primary myotube and C2C12 culture. In vitro investigation of the effects of GCs on postnatal myonuclear accretion showed significant inhibition by both corticosterone and synthetic GC dexamethasone (fold-change CORT = 0.5 *P* < 0.05; DEX = 0.3 *P* < 0.005; Fig. 5*G*).

These data demonstrate that 11βHSD1/KO animals have a greater GC response to LPS than WT counterparts in vivo. In addition, GCs can impair myonuclear accretion in vitro, therefore may impair muscle recovery following damage.

## DISCUSSION

Both enhanced systemic inflammation and elevated glucocorticoid responses have been implicated as important drivers of muscle wasting (17, 40–43) in acute exacerbations of COPD (AE-COPD) and pulmonary inflammation-induced muscle loss. 11 β-hydroxysteroid dehydrogenase type 1 (11β-HSD1) is a prereceptor regulator and gatekeeper of GC action, and its role in this context remains poorly defined. The aim of this study was to determine the contribution of 11β-HSD1 in muscle atrophy associated with acute exacerbations of COPD and test the hypothesis that its transgenic deletion in a mouse model of AE-COPD can abrogate GC-induced muscle wasting in this disease model setting.

Although the majority of AE-COPD is associated with a respiratory infection (44), the findings in our model utilizing the bacterial component LPS to induce pulmonary and systemic inflammation, require confirmation in models using other potential triggers of AE, such as viral pathogens to expand translatability to AE-COPD patients. We observed increased muscle atrophy in animals with global deletion of 11β-HSD1 following AE-COPD relative to their wild-type counterparts, characterized by increased measures of Ubiquitin 26S-proteasome (UPS) mediated degradation and suppressed markers of muscle anabolism and recovery. These findings were contrary to the original hypothesis based on murine models of corticosteroid excess, where we postulated that global deletion of 11β-HSD1 would protect from the anti-anabolic/catabolic actions of corticosteroids in muscle (45, 46). Instead, they more closely reflected observations of exacerbated muscle wasting previously reported in murine models of chronic inflammation with global deletion of 11β-HSD1, where the loss of local glucocorticoid reactivation fueled increased catabolic wasting (16). The most evident cause of the discrepancy between these studies may relate to the dosing of corticosteroid exposure, where 11β-HSD1 deletion protected from muscle wasting at higher therapeutic doses and exacerbated wasting when endogenous corticosteroid levels were examined. In this study, the increased muscle wasting observed in animals with transgenic deletion of 11β-HSD1 correlated with a marked increase in circulating endogenous corticosteroids and muscle GR signaling in response to AE-COPD.

Endogenous circulating GCs are significantly upregulated through the hypothalamic-pituitary-adrenal (HPA) axis, in response to various acute inflammatory stressors, including sepsis and the acute exacerbation of COPD (47, 48). Although elevated levels of endogenous circulating GCs support the suppression of systemic inflammation and improve survival outcomes (49), the increased activation of GC signaling in skeletal muscle drives proteolysis and reduced anabolic signaling. Previous studies have implicated the importance of GC signaling in mediating muscle atrophy in murine models of sepsis and inflammation-associated muscle wasting (50). Here, the targeted deletion of GR in mouse muscle showed prevention of muscle wasting in models of LPS-induced sepsis and in cancer-associated muscle wasting (50). Similarly, atrogene expression in rat muscle was blunted in response to LPS with inhibition of GR using RU-486 (51), further emphasizing the importance of GC signaling in skeletal muscle with inflammatory-associated muscle wasting.

The enzyme 11β-HSD1 mediates cellular GC action through its conversion of inactive GCs to their active counterparts (52). In prior studies, its transgenic deletion in models of corticosterone excess resulted in marked protection from GC-induced muscle wasting (including in models of inflammatory polyarthritis), preventing the activation of GC-induced catabolic pathways including the transcription factor FoxO1 and muscle-specific E3 ligases Atrogin-1 and MuRF-1 (45, 46). In contrast, its transgenic deletion in chronic models of TNF-α driven inflammatory polyarthritis in the absence of exogenous therapeutic GCs exacerbates inflammation-induced muscle wasting (16). These studies reveal the duality of the roles of 11β-HSD1 in muscle in inflammatory disease, where it mediates the muscle-protective anti-inflammatory properties of endogenous GCs, as well as mediating direct GC-induced muscle wasting in response to exogenous therapeutic GCs (16).

Although activation of the NF-κB pathway within muscle has been shown to be an important component of muscle wasting in following pulmonary inflammation (13), transgenic deletion of 11β-HSD1 did not enhance inflammatory signaling within the muscle (Supplemental Fig. S4) (13). These results would indicate that 11β-HSD1 appears to play a limited role in suppressing local muscle inflammation, and inflammatory muscle wasting in this model of AE-COPD. Systemic markers of inflammation showed a similar trend across both WT and 11BKO animals, with levels of IL-6 in response to LPS showing a comparable induction in both groups, suggesting that the severity of AE inflammation did not appear to mediate the differences in muscle wasting observed between WT and 11βHSD1/KO animals in this model (Supplemental Fig. S5).

However, although measures of inflammation were comparable between WT and 11βHSD1/KO animals, circulating levels of endogenous GCs showed a significant divergence with corticosterone being significantly elevated in 11βHSD1/KO animals following AE-COPD. This was accompanied by a significant increase in measures of GC signaling in muscle, with a marked increase in the response gene *Gilz* in 11βHSD1/KO animals (53). Together these results suggest that the muscles during AE-COPD, from 11βHSD1/KO animals are subjected to increased catabolic and antianabolic GC signaling as a result of elevated circulating GC exposure. Previously, elevated activation of the HPA axis and GC signaling within muscle have been shown to contribute to muscle atrophy in LPS-induced inflammation (8). This increased atrophy and GC response indicated that the HPA axis plays a pivotal role in driving inflammatory muscle catabolism. Proinflammatory mediators such as IL-6 and TNF-α are potent drivers of HPA axis activation and corticosteroid release (54). This process of HPA axis upregulation by proinflammatory mediators is in turn negatively regulated by circulating GCs in a negative feedback loop that facilitates the resolution of circulating GCs levels. 11β-HSD1 has been shown to be highly expressed, and dynamically regulated within the hypothalamus, with its local amplification of GCs playing a role in facilitating negative feedback of circulating corticosteroids in the HPA axis (55). This loss of 11β-HSD1 within the hypothalamus in 11βHSD1/KO animals may be one factor resulting in the elevated levels of circulating GCs and muscle wasting during AE-COPD in 11βHSD1/KO animals.

GCs induce muscle atrophy through several pathways, driving antianabolic (56, 57), and catabolic signaling primarily through activation of the UPS (36). Upon exposure to GCs, transcription factor FoxO1 increases in expression and activity (58), in turn, activating atrogenes such as Atrogin-1 and MuRF-1 (59). Increased FoxO1 transcript levels in primary human myotubes in response to cortisol confirm these effects of GCs, and the increases in FoxO1 protein levels in WT and 11βHSD1/KO muscle reflect preceding transcriptional GR actions induced by GCs. FoxO1 activity is subject to the regulation of its nuclear export following phosphorylation by Akt (39), and the kinetics of this inhibitory phosphorylation inversely correlate with muscle mass loss in response to pulmonary inflammation (60). We propose that increased circulating GCs may drive reduced inhibitory FoxO1 phosphorylation in AE-COPD in LPS-11βHSD1/KO animals contributing to the aggravated muscle loss we observe.

In line with previous observations in the related model of pulmonary inflammation, protein synthesis signaling through the IGF-1/Akt/mTOR pathway is reactivated 48 h following LPS in the muscle of WT animals, which signified initiation of muscle mass recovery in that study (60). Although mTOR activity was not directly measured in this study, increased phosphorylation of its indirect and direct downstream targets, i.e., S6, 4E-BP1, and ULK1 suggests activation of mTOR signaling in WT muscle. As the phosphorylation of Akt and the downstream mTOR targets is consistently lower or absent, this suggests attenuated protein synthesis signaling in muscle of 11βHSD1/KO compared with WT animals following AE-COPD. Such anti-anabolic effects of GCs on muscle by antagonism of the IGF-1/Akt/mTOR pathway have been well established (56, 57). In vivo treatment of methylprednisolone, a synthetic GC, in male rats showed significant reductions in IGF-1 mRNA expression in the gastrocnemius (61). It has been previously demonstrated that Akt signaling is impaired by nongenomic actions of GR by GCs, which block IRS-1-PI3K interactions, resulting in reduced Akt phosphorylation and suppression of downstream mTOR signaling (62). Although phosphorylation of S6 kinase and 4E-BP1 are instrumental for initiating mRNA translation, GC treatment of myoblasts significantly reduces S6 phosphorylation, illustrating the direct inhibitory actions of GCs on protein synthesis (56). Taken together, we speculate attenuated anabolic signaling in LPS-11βHSD1/KO mice compared with WT counterparts reflects the impact of residual elevations in circulating GCs and contributes to reduced muscle mass. Further measures of muscle anabolism, such as assessment of protein synthesis using puromycin incorporation are required to effectively validate these observations (63).

Another process involved in muscle mass maintenance and recovery is postnatal myogenesis, in which satellite cells differentiate and fuse with existing fibers (64). Myogenesis has been shown to be inhibited by systemic cues, including inflammatory cytokines and GCs (65), resulting in reduced satellite cell proliferation and differentiation (66), and myonuclear accretion (67). We modeled postnatal myogenesis-driven muscle recovery in vitro to assess the presence of circulating mediators in plasma that impact this process. Myonuclear accretion in response to a standardized atrophic stimulus has a trend toward attenuation in presence of plasma from LPS-11βHSD1/KO mice compared with WT counterparts. Although no difference in inflammatory cytokine IL-6 levels is present (Supplemental Fig. S5), the significant increases in corticosterone plasma levels suggest GCs directly interfere with myonuclear accretion. In line with this notion, treatment with corticosterone or dexamethasone is sufficient to inhibit myonuclear accretion. Although not assessed in a postnatal myogenesis set-up as done here, recent studies have shown inhibition of myogenesis in C2C12 cells following dexamethasone and cortisone treatment, in line with our findings (68). Combined, the elevated circulating GCs levels present in LPS-11βHSD1/KO mice may be responsible for suppressed protein synthesis signaling and impaired myonuclear accretion, contributing to exaggerated muscle wasting.

In this study, we deployed a murine model of AE-COPD driven by repeated elastase instillation and LPS induction of pulmonary inflammation, characterized by emphysema, pulmonary and systemic inflammation, and muscle atrophy (29). Global deletion of 11β-HSD1 allows for translational extrapolation to the use of therapeutic 11β-HSD1 inhibitors. However, our study indicated that this approach does not prevent muscle wasting elicited by AE-COPD. However, a targeted, muscle-specific 11β-HSD1/KO approach would help disentangle deleterious and positive actions of both local and systemic actions of 11β-HSD1.

This study highlights the important role of 11β-HSD1 in mediating muscle wasting during an acute exacerbation of COPD. We show that with the transgenic deletion of 11β-HSD1 during AE-COPD, there is a compensatory increase in circulating corticosterone during the resolution phase of pulmonary and systemic inflammation, which correlates with sustained activation of UPS-mediated proteolysis and reduced muscle anabolic signaling. Based on these findings, the use of therapeutic 11β-HSD1 inhibitors during an acute exacerbation of COPD may not be appropriate in this setting. This study shows that 11β-HSD1 inhibition does not prevent but aggravates muscle wasting during an acute exacerbation of COPD.

## DATA AVAILABILITY

Data will be made available upon reasonable request.

## SUPPLEMENTAL DATA

10.6084/m9.figshare.21187471 Supplemental Fig. S1: https://doi.org/10.6084/m9.figshare.21187471.

10.6084/m9.figshare.21187489 Supplemental Fig. S2: https://doi.org/10.6084/m9.figshare.21187489.

10.6084/m9.figshare.21187507 Supplemental Fig. S3: https://doi.org/10.6084/m9.figshare.21187507.

10.6084/m9.figshare.21187573 Supplemental Fig. S4: https://doi.org/10.6084/m9.figshare.21187573.

10.6084/m9.figshare.21187582 Supplemental Fig. S5: https://doi.org/10.6084/m9.figshare.21187582.

10.6084/m9.figshare.21187570 Supplemental Fig. S6: https://doi.org/10.6084/m9.figshare.21187570.

## GRANTS

This research was supported by Versus Arthritis Grants (Reference: 19859 and 20843).

## DISCLOSURES

No conflicts of interest, financial or otherwise, are declared by the authors.

## AUTHOR CONTRIBUTIONS

F.V., G.G.L., A.M.W.J.S., R.S.H., and R.C.J.L. conceived and designed research; J.M.W., K.W., W.R.P.H.v.d.W., M.C.J.M.K., S.L., C.M., and R.C.J.L. performed experiments; J.M.W., K.W., M.C.J.M.K., C.M., B.V.d.H., C.S., R.S.H., and R.C.J.L. analyzed data; J.M.W., K.W., C.M., R.S.H., and R.C.J.L. interpreted results of experiments; J.M.W., K.W., B.V.d.H., R.S.H., and R.C.J.L. prepared figures; J.M.W. drafted manuscript; J.M.W., W.R.P.H.v.d.W., M.C.J.M.K., S.L., C.M., F.V., B.V.d.H., C.S., R.S.H., and R.C.J.L. edited and revised manuscript; J.M.W., G.G.L., A.M.W.J.S., R.S.H., and R.C.J.L. approved final version of manuscript.
